# (4*Z*,6*Z*,12*Z*,14*Z*)-2,10-Dimethyl-2,8,10,16-tetra­hydro­dipyrazolo[3,4-*e*:3′,4′-*l*][1,2,4,8,9,11]hexa­azacyclo­tetra­decine-4,12-diamine

**DOI:** 10.1107/S1600536809022132

**Published:** 2009-06-13

**Authors:** Anton V. Dolzhenko, Giorgia Pastorin, Anna V. Dolzhenko, Geok Kheng Tan, Lip Lin Koh

**Affiliations:** a Department of Pharmacy, Faculty of Science, National University of Singapore, 18 Science Drive 4, Singapore 117543, Singapore; b Department of Chemistry, Faculty of Science, National University of Singapore, 3 Science Drive 3, Singapore 117543, Singapore

## Abstract

The title compound, C_12_H_16_N_12_, is a centrosymmetric mol­ecule which comprises of a hexa­aza[14]annulene macrocyclic ring fused with two pyrazole rings. The macrocyclic ring is essentially planar, with an r.m.s. deviation of 0.0381 Å. The electron pairs of the amino groups are delocalized with the conjugated system of the macrocycle. Strong intra­molecular N—H⋯N hydrogen bonds arranged in an *S*_2_^2^(10) graph-set motif are present in the macrocyclic ring. In the crystal, the amino groups act as donors for inter­molecular N—H⋯N inter­actions with the N atoms of the heterocyclic system, forming a network of two types of extended chains oriented parallel to the [101] and [011] directions. The crystal packing is also stabilized by weak inter­molecular C—H⋯N hydrogen bonds formed between pyrazole C—H groups and N atoms of the macrocyclic ring, running in the [10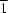] direction.

## Related literature

The title compound was synthesized according to Dolzhenko *et al.* (2009 ▶). For the synthesis and crystal structure studies of related macrocyclic compounds (as nickel complexes), see: Gradinaru *et al.* (2001 ▶); Gerbeleu *et al.* (1991 ▶); Leovac *et al.* (1993 ▶) and references cited therein; Simonov *et al.* (1988 ▶). For a review of the graph-set analysis of hydrogen bonding, see: Bernstein *et al.* (1995 ▶).
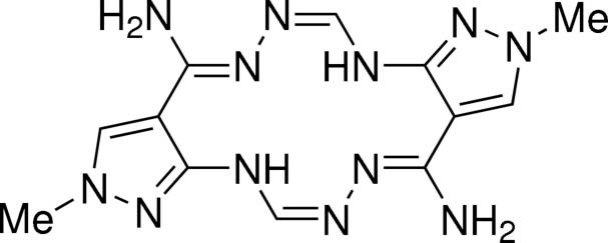


## Experimental

### 

#### Crystal data

C_12_H_16_N_12_
*M* *_r_* = 328.37
Monoclinic, 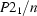

*a* = 7.1470 (6) Å
*b* = 7.5593 (7) Å
*c* = 13.9174 (13) Å
β = 91.866 (3)°
*V* = 751.51 (12) Å^3^
*Z* = 2
Mo *K*α radiation
μ = 0.10 mm^−1^
*T* = 223 K
0.22 × 0.08 × 0.06 mm

#### Data collection

Bruker SMART APEX CCD diffractometer
Absorption correction: multi-scan (*SADABS*; Sheldrick, 2001 ▶) *T*_min_ = 0.978, *T*_max_ = 0.994
5126 measured reflections
1721 independent reflections
1272 reflections with *I* > 2σ(*I*)
*R*_int_ = 0.037

#### Refinement

*R*[*F* ^2^ > 2σ(*F* ^2^)] = 0.062
*wR*(*F* ^2^) = 0.151
*S* = 1.06
1721 reflections
122 parameters
H atoms treated by a mixture of independent and constrained refinement
Δρ_max_ = 0.26 e Å^−3^
Δρ_min_ = −0.17 e Å^−3^

### 

Data collection: *SMART* (Bruker, 2001 ▶); cell refinement: *SAINT* (Bruker, 2001 ▶); data reduction: *SAINT*; program(s) used to solve structure: *SHELXS97* (Sheldrick, 2008 ▶); program(s) used to refine structure: *SHELXL97* (Sheldrick, 2008 ▶); molecular graphics: *SHELXTL* (Sheldrick, 2008 ▶); software used to prepare material for publication: *SHELXTL*.

## Supplementary Material

Crystal structure: contains datablocks I, global. DOI: 10.1107/S1600536809022132/si2181sup1.cif

Structure factors: contains datablocks I. DOI: 10.1107/S1600536809022132/si2181Isup2.hkl

Additional supplementary materials:  crystallographic information; 3D view; checkCIF report

## Figures and Tables

**Table 1:** Hydrogen-bond geometry (Å, °)

*D*—H⋯*A*	*D*—H	H⋯*A*	*D*⋯*A*	*D*—H⋯*A*
N3—H3*N*⋯N4	0.85 (3)	2.09 (3)	2.770 (3)	136 (2)
N5—H5*A*⋯N1^i^	0.88 (3)	2.33 (3)	3.065 (3)	141 (2)
N5—H5*B*⋯N6^ii^	0.85 (3)	2.61 (3)	3.466 (3)	174 (2)
C3—H3⋯N6^ii^	0.94	2.51	3.402 (3)	158

Symmetry codes: (i) 
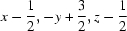
; (ii) 
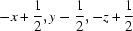
.

**Fig. 1.**
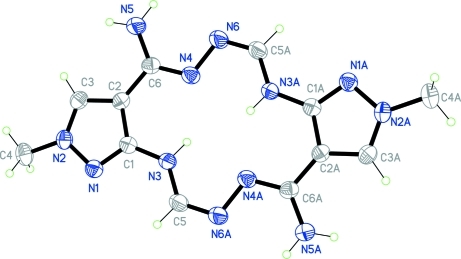
The molecular structure of the title compound with the atomic numbering scheme. Displacement ellipsoids are drawn at the 50% probability level.

**Fig. 2.**
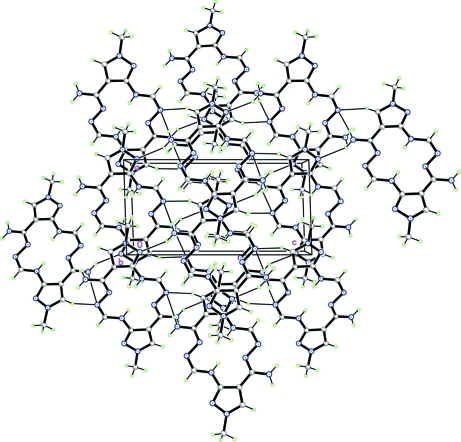
Molecular parking in the crystal, viewed along the b axis.

## supplementary crystallographic information

### Comment

Macrocyclic compounds, various classes of which have been known for a long time, attract significant attention of the chemists' community due to their unique physico-chemical properties. Several studies on the synthesis and the crystal structure of the planar 1,2,4,8,9,11-hexaaza[14]annulene macrocyclic system have appeared (Gradinaru *et al.*, 2001; Gerbeleu *et al.*, 1991; Leovac *et al.*, 1993; Simonov *et al.*, 1988). However, all of the compounds were prepared and investigated as nickel complexes. Herein, we report the first crystal structure of the ligand with the 1,2,4,8,9,11-hexaaza[14]annulene macrocyclic ring. The title compound is a centrosymmetric molecule, which comprises the hexaaza[14]annulene macrocyclic ring fused with two pyrazole rings. The macrocyclic ring is essentially planar with an r.m.s. deviation of 0.0381 Å. The most outlying from the least-squares plane of the marcocyclic ring are atoms C6 (C6A) and N4 (N4A) with deviations of 0.0654 (17) Å and 0.0577 (18) Å, respectively. The C6—N5 bond distance (1.340 (3) Å) indicates delocalization of the electron pair of the N5 atom, though the amino group adopts a slightly pyramidal geometry with 0.084 (15) Å deviation of N5 from the C6/H5A/H5B mean plane. The strong intramolecular N—H···N hydrogen bonds arranged in a *S*^2^_2_(10) graph-set motif (Bernstein *et al.*, 1995) are present inside the macrocyclic ring.

In the crystal, the amino groups act as donors for intermolecular N—H···N interaction with the nitrogen atoms N1 (N1A) and N6 (N6A), thereby forming two types of extended chains. The nitrogen atoms N1 (N1A) are acceptors in *C*(6) chains running parallel to the [101] direction, while the nitrogen atoms N6 (N6A) are acceptors in *C*(5) chains oriented in the [011] direction. Together, these hydrogen bonds form a centrosymmetric *R*^4^_4_(22) motif. The crystal packing is also stabilized by weak intermolecular C—H···N hydrogen bonds, formed between C3—H (C3A—H) of the pyrazole rings and nitrogen atoms N6 (N6A) of the macrocyclic ring, running in the [101] direction.

### Experimental

The title compound was synthesized according to Dolzhenko *et al.* (2009). Single crystals suitable for crystallographic analysis were grown by recrystallization from methanol.

### Refinement

All the H atoms attached to the carbon atoms were constrained in a riding motion approximation [0.94 Å for C_aryl_—H and 0.97 Å for methyl groups; *U*_iso_(H) =1.2*U*_eq_(C_aryl_) and *U*_iso_(H) =1.5*U*_eq_(C_methyl_)] while the N-bound H atoms were located in a difference map and refined freely.

### Crystal data

**Table:**

C_12_H_16_N_12_	*F*(000) = 344
*M**_r_* = 328.37	*D*_x_ = 1.451 Mg m^−^^3^
Monoclinic, *P*2_1_/*n*	Melting point: 537 K
Hall symbol: -P 2yn	Mo *K*α radiation, λ = 0.71073 Å
*a* = 7.1470 (6) Å	Cell parameters from 734 reflections
*b* = 7.5593 (7) Å	θ = 2.9–21.6°
*c* = 13.9174 (13) Å	µ = 0.10 mm^−^^1^
β = 91.866 (3)°	*T* = 223 K
*V* = 751.51 (12) Å^3^	Block, colourless
*Z* = 2	0.22 × 0.08 × 0.06 mm

### Data collection

**Table:**

Bruker SMART APEX CCD diffractometer	1721 independent reflections
Radiation source: fine-focus sealed tube	1272 reflections with *I* > 2σ(*I*)
graphite	*R*_int_ = 0.037
φ and ω scans	θ_max_ = 27.5°, θ_min_ = 2.9°
Absorption correction: multi-scan (*SADABS*; Sheldrick, 2001)	*h* = −9→8
*T*_min_ = 0.978, *T*_max_ = 0.994	*k* = −8→9
5126 measured reflections	*l* = −18→15

### Refinement

**Table:**

Refinement on *F*^2^	Primary atom site location: structure-invariant direct methods
Least-squares matrix: full	Secondary atom site location: difference Fourier map
*R*[*F*^2^ > 2σ(*F*^2^)] = 0.062	Hydrogen site location: inferred from neighbouring sites
*wR*(*F*^2^) = 0.151	H atoms treated by a mixture of independent and constrained refinement
*S* = 1.06	*w* = 1/[σ^2^(*F*_o_^2^) + (0.0676*P*)^2^ + 0.2208*P*] where *P* = (*F*_o_^2^ + 2*F*_c_^2^)/3
1721 reflections	(Δ/σ)_max_ = 0.002
122 parameters	Δρ_max_ = 0.26 e Å^−^^3^
0 restraints	Δρ_min_ = −0.17 e Å^−^^3^

### Special details

**Table:**

Geometry. All e.s.d.'s (except the e.s.d. in the dihedral angle between two l.s. planes) are estimated using the full covariance matrix. The cell e.s.d.'s are taken into account individually in the estimation of e.s.d.'s in distances, angles and torsion angles; correlations between e.s.d.'s in cell parameters are only used when they are defined by crystal symmetry. An approximate (isotropic) treatment of cell e.s.d.'s is used for estimating e.s.d.'s involving l.s. planes.
Refinement. Refinement of *F*^2^ against ALL reflections. The weighted *R*-factor *wR* and goodness of fit *S* are based on *F*^2^, conventional *R*-factors *R* are based on *F*, with *F* set to zero for negative *F*^2^. The threshold expression of *F*^2^ > σ(*F*^2^) is used only for calculating *R*-factors(gt) *etc*. and is not relevant to the choice of reflections for refinement. *R*-factors based on *F*^2^ are statistically about twice as large as those based on *F*, and *R*- factors based on ALL data will be even larger.

### Fractional atomic coordinates and isotropic or equivalent isotropic displacement parameters (Å^2^)

**Table:**

	*x*	*y*	*z*	*U*_iso_*/*U*_eq_	
N1	0.5186 (2)	0.7646 (2)	0.56112 (13)	0.0318 (5)	
N2	0.6151 (3)	0.6882 (2)	0.48829 (14)	0.0331 (5)	
N3	0.2241 (3)	0.9124 (3)	0.56302 (13)	0.0330 (5)	
H3N	0.135 (4)	0.945 (3)	0.5254 (18)	0.041 (7)*	
N4	0.0731 (2)	0.9323 (2)	0.37750 (13)	0.0329 (5)	
N5	0.2238 (4)	0.7748 (3)	0.25680 (16)	0.0483 (6)	
H5A	0.125 (4)	0.790 (4)	0.218 (2)	0.050 (8)*	
H5B	0.312 (4)	0.702 (3)	0.2455 (19)	0.040 (7)*	
N6	−0.0650 (3)	0.9742 (3)	0.30609 (14)	0.0417 (5)	
C1	0.3648 (3)	0.8253 (3)	0.51677 (15)	0.0286 (5)	
C2	0.3588 (3)	0.7909 (3)	0.41682 (15)	0.0291 (5)	
C3	0.5250 (3)	0.7009 (3)	0.40344 (16)	0.0331 (5)	
H3	0.5666	0.6566	0.3448	0.040*	
C4	0.8014 (3)	0.6185 (3)	0.50829 (19)	0.0416 (6)	
H4A	0.8443	0.5566	0.4521	0.062*	
H4B	0.7981	0.5372	0.5621	0.062*	
H4C	0.8863	0.7151	0.5240	0.062*	
C5	0.2062 (3)	0.9460 (3)	0.65708 (17)	0.0381 (6)	
H5	0.3035	0.9088	0.6994	0.046*	
C6	0.2100 (3)	0.8348 (3)	0.34691 (15)	0.0305 (5)	

### Atomic displacement parameters (Å^2^)

**Table:**

	*U*^11^	*U*^22^	*U*^33^	*U*^12^	*U*^13^	*U*^23^
N1	0.0292 (10)	0.0340 (10)	0.0322 (10)	−0.0006 (8)	0.0010 (8)	0.0006 (8)
N2	0.0271 (10)	0.0347 (10)	0.0378 (11)	0.0028 (8)	0.0043 (8)	0.0001 (8)
N3	0.0293 (10)	0.0434 (11)	0.0264 (10)	0.0052 (9)	−0.0009 (8)	−0.0012 (9)
N4	0.0288 (10)	0.0427 (10)	0.0272 (9)	0.0007 (8)	0.0014 (8)	0.0031 (8)
N5	0.0422 (14)	0.0694 (16)	0.0330 (12)	0.0178 (12)	−0.0036 (10)	−0.0110 (11)
N6	0.0367 (11)	0.0604 (13)	0.0279 (10)	0.0100 (10)	0.0002 (9)	0.0016 (10)
C1	0.0263 (11)	0.0294 (10)	0.0301 (11)	−0.0045 (9)	0.0009 (9)	0.0016 (9)
C2	0.0294 (11)	0.0293 (10)	0.0288 (11)	−0.0029 (9)	0.0045 (9)	−0.0006 (9)
C3	0.0332 (12)	0.0340 (11)	0.0324 (12)	−0.0010 (10)	0.0064 (10)	−0.0010 (10)
C4	0.0289 (12)	0.0424 (13)	0.0536 (15)	0.0047 (10)	0.0023 (11)	0.0050 (12)
C5	0.0341 (13)	0.0511 (14)	0.0291 (12)	0.0049 (11)	−0.0014 (10)	0.0035 (11)
C6	0.0304 (12)	0.0343 (11)	0.0269 (11)	−0.0038 (9)	0.0035 (9)	0.0003 (9)

### Geometric parameters (Å, °)

**Table:**

N1—C1	1.325 (3)	N5—H5B	0.85 (3)
N1—N2	1.372 (3)	N6—C5^i^	1.295 (3)
N2—C3	1.330 (3)	C1—C2	1.414 (3)
N2—C4	1.450 (3)	C2—C3	1.387 (3)
N3—C5	1.344 (3)	C2—C6	1.456 (3)
N3—C1	1.379 (3)	C3—H3	0.9400
N3—H3N	0.85 (3)	C4—H4A	0.9700
N4—C6	1.307 (3)	C4—H4B	0.9700
N4—N6	1.413 (3)	C4—H4C	0.9700
N5—C6	1.340 (3)	C5—N6^i^	1.295 (3)
N5—H5A	0.88 (3)	C5—H5	0.9400
			
C1—N1—N2	103.35 (18)	C1—C2—C6	127.84 (19)
C3—N2—N1	112.65 (18)	N2—C3—C2	108.0 (2)
C3—N2—C4	127.7 (2)	N2—C3—H3	126.0
N1—N2—C4	119.45 (19)	C2—C3—H3	126.0
C5—N3—C1	129.7 (2)	N2—C4—H4A	109.5
C5—N3—H3N	116.9 (17)	N2—C4—H4B	109.5
C1—N3—H3N	113.3 (17)	H4A—C4—H4B	109.5
C6—N4—N6	114.22 (18)	N2—C4—H4C	109.5
C6—N5—H5A	116.4 (17)	H4A—C4—H4C	109.5
C6—N5—H5B	117.8 (18)	H4B—C4—H4C	109.5
H5A—N5—H5B	124 (2)	N6^i^—C5—N3	125.0 (2)
C5^i^—N6—N4	111.17 (19)	N6^i^—C5—H5	117.5
N1—C1—N3	123.7 (2)	N3—C5—H5	117.5
N1—C1—C2	113.13 (19)	N4—C6—N5	125.0 (2)
N3—C1—C2	123.2 (2)	N4—C6—C2	116.68 (19)
C3—C2—C1	102.91 (19)	N5—C6—C2	118.3 (2)
C3—C2—C6	129.2 (2)		

Symmetry codes: (i) −*x*, −*y*+2, −*z*+1.

### Hydrogen-bond geometry (Å, °)

**Table:**

*D*—H···*A*	*D*—H	H···*A*	*D*···*A*	*D*—H···*A*
N3—H3N···N4	0.85 (3)	2.09 (3)	2.770 (3)	136 (2)
N5—H5A···N1^ii^	0.88 (3)	2.33 (3)	3.065 (3)	141 (2)
N5—H5B···N6^iii^	0.85 (3)	2.61 (3)	3.466 (3)	174 (2)
C3—H3···N6^iii^	0.94	2.51	3.402 (3)	158

Symmetry codes: (ii) *x*−1/2, −*y*+3/2, *z*−1/2; (iii) −*x*+1/2, *y*−1/2, −*z*+1/2.
